# Infections, vaccinations, and the risk of childhood leukaemia

**DOI:** 10.1038/sj.bjc.6690548

**Published:** 1999-07

**Authors:** J D Dockerty, D C G Skegg, J M Elwood, G P Herbison, D M O Becroft, M E Lewis

**Affiliations:** Childhood Cancer Research Group, University of Oxford, 57 Woodstock Road, Oxford, OX2 6HJ, UK; Department of Preventive and Social Medicine, University of Otago, PO Box 913, Dunedin, New Zealand; Department of Obstetrics and Gynaecology, University of Auckland, National Women's Hospital, Claude Road, Epsom, Auckland, New Zealand; Department of Paediatrics, Wellington School of Medicine, PO Box 7343, Wellington South, New Zealand

**Keywords:** epidemiology, leukaemia, infection, vaccination, case-control studies, influenza

## Abstract

A nationwide case-control study was conducted in New Zealand, to test hypotheses about the role of infections in the aetiology of childhood leukaemia. Children aged 0–14 years with leukaemia were matched on age and sex to controls selected from birth records. Case ascertainment was virtually complete and 121 (92%) of 131 eligible case families took part. The participation rate among the 303 first-choice eligible controls was 69%. Home interviews and serological tests were conducted. Adjusted relative risks were estimated by logistic regression. There was an increased risk of leukaemia in relation to reported influenza infection of the child during the first year of life (adjusted odds ratio 6.8, 95% confidence interval 1.8–25.7). This could be a chance finding due to multiple comparisons, and it should be tested elsewhere. Some key variables relevant to Greaves' hypothesis were not associated with B-cell precursor acute lymphoblastic leukaemia (numbers of infections and vaccinations, firstborn status, attendance at preschool groups), although a small effect could not be ruled out with a study of this size. Leukaemia risk was higher among children in poorer social circumstances, and this was true for all eligible children as well as for the participants. © 1999 Cancer Research Campaign


*British Journal of Cancer* (1999) **80**(9), 1483–1489
© 1999 Cancer Research Campaign
Article no. bjoc.1999.0548

### Infections, vaccinations, and the risk of childhood leukaemia

**JD Dockerty[1,2], DCG Skegg[2], JM Elwood[2], GP Herbison[2], DMO Becroft[3] and ME Lewis[4]**

[1]Childhood Cancer Research Group, University of Oxford, 57 Woodstock Road, Oxford OX2 6HJ, UK; [2]Department of Preventive and Social Medicine, University of Otago, PO Box 913, Dunedin, New Zealand; [3]Department of Obstetrics and Gynaecology, University of Auckland, National Women's Hospital, Claude Road, Epsom, Auckland, New Zealand; [4]Department of Paediatrics, Wellington School of Medicine, PO Box 7343, Wellington South, New Zealand

**Summary** A nationwide case-control study was conducted in New Zealand, to test hypotheses about the role of infections in the aetiology of childhood leukaemia. Children aged 0–14 years with leukaemia were matched on age and sex to controls selected from birth records. Case ascertainment was virtually complete and 121 (92%) of 131 eligible case families took part. The participation rate among the 303 first-choice eligible controls was 69%. Home interviews and serological tests were conducted. Adjusted relative risks were estimated by logistic regression. There was an increased risk of leukaemia in relation to reported influenza infection of the child during the first year of life (adjusted odds ratio 6.8, 95% confidence interval 1.8–25.7). This could be a chance finding due to multiple comparisons, and it should be tested elsewhere. Some key variables relevant to Greaves' hypothesis were not associated with B-cell precursor acute lymphoblastic leukaemia (numbers of infections and vaccinations, firstborn status, attendance at preschool groups), although a small effect could not be ruled out with a study of this size. Leukaemia risk was higher among children in poorer social circumstances, and this was true for all eligible children as well as for the participants.

**Keywords:** epidemiology; leukaemia; infection; vaccination; case-control studies; influenza

For many years it has been suspected that one or more viruses might cause leukaemia in children. Kinlen (1988) suggested that childhood leukaemia occurs as a rare response to a specific (and common) infectious agent, and that population influxes into rural areas favour epidemics of the infection and increases in leukaemia incidence. Greaves (1988) suggested that the risk of the common B-cell precursor subtype of acute lymphoblastic leukaemia (ALL) was increased by delayed or diminished exposure to infections in infancy. Supporting evidence for an infective origin of childhood leukaemia has come from descriptive, clustering and case-control studies (Greaves and Alexander, 1993; Kinlen, 1996, 1997). The hypotheses need further testing in analytical studies (Neglia et al, 1990). In this national, population-based case-control study, the hypotheses were: (1) that children infected with one or more specific viruses are at increased risk of childhood leukaemia; and (2) that children with diminished or delayed exposure to common infections in infancy are at increased risk of ALL.

### MATERIALS AND METHODS

Details of case ascertainment, pathology review, control selection and recruitment procedures have already been described (Dockerty et al, 1998). The 131 eligible cases were newly diagnosed with childhood leukaemia (ages 0–14 years) 1990–1993, and born and resident in New Zealand (NZ). Controls (matched 1:1 to cases on age and sex) were selected randomly from the NZ-born and resident childhood population, using national birth records. Each control's birth was registered in the same quarter of the same year as the matched case. Adopted children were not eligible.

Maternal interviews were available for 121 children with leukaemia (92%); there were eight refusals, one notification by death certificate only (not contacted) and one questionnaire lost by a courier. The age distribution of the 121 was: 69 aged 0–4 years, 34 aged 5–9 years and 18 aged 10–14 years. Of the 121 matched first-choice controls, 89 (74%) took part; there were 11 refusals, 19 not found, one mother with language difficulties and one mother was ill. Replacement controls were found for the 32 non-participants using the same methods.

The wider study also included 213 solid cancer cases with additional age- and sex-matched controls (Dockerty et al, 1998), giving a total of 303 available controls who could contribute to unconditional logistic regressions (see Analyses). Of all 303 first-choice controls, the mothers of 209 (69%) were interviewed. The 94 who did not take part included 46 not found, 46 refusals, one with language difficulties, and one ill mother.

The mothers were interviewed in their homes during 1991–1995, with identical standardized questionnaires for cases and controls. Neither parents nor interviewers were informed about the hypotheses. The questionnaire was adapted from that of Drs Patricia McKinney and Eve Roman in the UK. Vaccination histories were supplemented with information from parent-held 'Health and Development' records. A 'reference date' was used. For a case, this was the date of diagnosis. For a control, it was the date on which the child achieved the exact age the matched case was at diagnosis. The child's social class (Office of Population Censuses and Surveys, 1991) was derived as the higher of the mother's and father's. 'Household density' was the average

*Received 14 December 1998*
*Revised 7 January 1999*
*Accepted 12 January 1999*

*Correspondence to:* JD Dockerty, Childhood Cancer Research Group, University of Oxford, 57 Woodstock Road, Oxford OX2 6HJ, UK

**Table 1** Odds ratios for demographic factors and variables listed primarily as possible confounders. The odds ratios in this table are adjusted for age (in years) and sex

| | | Childhood leukaemias | | | | | Acute lymphoblastic leukaemia | | | | |
|---|---|---|---|---|---|---|---|---|---|---|---|
| | | | | | 95% CI Bounds | | | | | 95% CI Bounds | |
| Characteristic or exposure | Categories | No. of cases | No. of controls | Odds ratio | Lower | Upper | No. of cases | No. of controls | Odds ratio | Lower | Upper |
| Child's social class (highest of parents) | I or II | 40 | 145 | 1.00 | | | 34 | 145 | 1.00 | | |
| | IIIN or IIIM | 41 | 90 | 1.66 | 0.98 | 2.81 | 35 | 90 | 1.65 | 0.94 | 2.91 |
| | IV or V | 13 | 32 | 1.48 | 0.69 | 3.15 | 8 | 32 | 1.04 | 0.43 | 2.52 |
| | No paid jobs – not classifiable | 26 | 32 | 3.00 | 1.57 | 5.71 | 19 | 32 | 2.69 | 1.32 | 5.48 |
| Was mother married at time of interview? | No | 47 | 70 | 1.00 | | | 36 | 70 | 1.00 | | |
| | Yes | 74 | 233 | 0.44 | 0.27 | 0.70 | 61 | 233 | 0.48 | 0.29 | 0.80 |
| Child's ethnic group | Non-Maori | 104 | 269 | 1.00 | | | 85 | 269 | 1.00 | | |
| | Maori | 17 | 33 | 1.42 | 0.74 | 2.74 | 12 | 33 | 1.23 | 0.59 | 2.58 |
| Mother's highest educational qualification | No qualifications | 45 | 65 | 1.00 | | | 31 | 65 | 1.00 | | |
| | School Certificate | 15 | 62 | 0.28 | 0.14 | 0.57 | 13 | 62 | 0.34 | 0.16 | 0.74 |
| | 6th form Cert to Scholarship | 15 | 34 | 0.59 | 0.28 | 1.25 | 13 | 34 | 0.73 | 0.32 | 1.64 |
| | Post-school non-University | 32 | 115 | 0.37 | 0.21 | 0.65 | 27 | 115 | 0.44 | 0.23 | 0.83 |
| | University | 14 | 27 | 0.68 | 0.31 | 1.47 | 13 | 27 | 0.87 | 0.38 | 1.99 |
| Mother-home owner? | No | 40 | 77 | 1.00 | | | 29 | 77 | 1.00 | | |
| | Yes | 79 | 226 | 0.63 | 0.39 | 1.03 | 66 | 226 | 0.74 | 0.43 | 1.27 |
| Child's gestational age | 40 weeks | 59 | 138 | 1.00 | | | 50 | 138 | 1.00 | | |
| | 29–39 weeks | 35 | 73 | 0.99 | 0.58 | 1.67 | 29 | 73 | 0.93 | 0.53 | 1.65 |
| | 41–45 weeks | 26 | 92 | 0.67 | 0.39 | 1.17 | 18 | 92 | 0.53 | 0.28 | 0.99 |
| Mother's age at birth of child | 15–34 yrs | 107 | 279 | 1.00 | | | 84 | 279 | 1.00 | | |
| | 35–41 yrs | 14 | 24 | 1.56 | 0.74 | 3.29 | 13 | 24 | 1.88 | 0.86 | 4.14 |
| Child's birth weight | 3000–3999 g | 84 | 212 | 1.00 | | | 67 | 212 | 1.00 | | |
| | under 3000 g | 19 | 52 | 0.89 | 0.49 | 1.63 | 16 | 52 | 0.93 | 0.49 | 1.78 |
| | 4000 g or more | 16 | 36 | 1.05 | 0.53 | 2.07 | 12 | 36 | 0.90 | 0.42 | 1.91 |
| Mother had any X-rays during index pregnancy or 3 months before? | No | 103 | 251 | 1.00 | | | 83 | 251 | 1.00 | | |
| | Yes | 13 | 37 | 0.87 | 0.44 | 1.74 | 9 | 37 | 0.75 | 0.34 | 1.67 |
| Mother had any abdominal or pelvic X-rays during pregnancy or 3 months before? | No | 108 | 273 | 1.00 | | | 87 | 273 | | | |
| | Yes | 8 | 15 | 1.56 | 0.62 | 3.97 | 5 | 15 | 1.24 | 0.41 | 3.69 |
| Child: any X-rays/ radiotherapy before reference date?[a] | No | 67 | 178 | 1.00 | | | 55 | 178 | 1.00 | | |
| | Yes | 53 | 123 | 1.20 | 0.76 | 1.90 | 42 | 123 | 1.15 | 0.69 | 1.89 |
| Did mother smoke cigarettes in pregnancy or 3 months before? | No | 71 | 203 | 1.00 | | | 56 | 203 | 1.00 | | |
| | Yes | 49 | 99 | 1.54 | 0.97 | 2.42 | 40 | 99 | 1.66 | 1.01 | 2.74 |
| Did mother ever regularly smoke cigarettes (> 1 per day)? | No | 47 | 140 | 1.00 | | | 37 | 140 | 1.00 | | |
| | Yes | 74 | 163 | 1.43 | 0.92 | 2.22 | 60 | 163 | 1.50 | 0.92 | 2.43 |

[a]X-rays within 3 months of the reference date are excluded.

number of people per room in the house the child lived in at birth. 'Residential mobility' was the number of houses the child ever lived in up to the reference date.

Undetected cancer could alter some exposures among cases before diagnosis. Because such changes in exposure would be a consequence of the illness, it would be wrong to consider them in the analyses. Thus, X-rays within 3 months of the reference date, and vaccinations in or after the calendar year of the diagnosis/ reference date were not counted as exposures for cases or controls. Where the exposure period of interest was the first 12 months of life (e.g. infections), children aged under 15 months on the reference date were excluded. Specific vaccinations were counted only if the year when they were given was known and was before the year of the reference date. Children aged under *n* months on the reference date were excluded from the analyses for a particular vaccination, where *n* was 3 months plus the youngest age when the vaccination would be given.

### Analyses

Analyses were conducted for combined leukaemias (121) and ALL (97 cases). Unconditional logistic regressions in SPSS included these cases and all 303 controls. Such an approach, in a study with matching only on age and sex, should be valid given that the matched factors were adjusted for (Rothman, 1986; Hamajima et al, 1994). We checked whether there was any important bias towards a null effect (Breslow and Day, 1980) by repeating the ALL analyses with conditional logistic regression (97 matched pairs, EGRET software).

 *© 1999 Cancer Research Campaign*

**Table 2** Odds ratios for childhood leukaemias in relation to infections of the mother during her pregnancy with the child, or in the 3 months before the pregnancy

| Infection[a] | Categories | No. of cases | No. of controls | Adjusted for age in years and sex only: Odds ratio | 95% CI Bounds: Lower | 95% CI Bounds: Upper | Adjusted for age, sex, and other variables[c]: Odds ratio | 95% CI Bounds: Lower | 95% CI Bounds: Upper |
|---|---|---|---|---|---|---|---|---|---|
| Influenza | No | 111 | 271 | 1.00 | | | 1.00 | | |
| | Yes | 8 | 27 | 0.62 | 0.27 | 1.45 | 0.58 | 0.24 | 1.41 |
| Cystitis or kidney infection | No | 117 | 287 | 1.00 | | | 1.00 | | |
| | Yes | 3 | 13 | 0.50 | 0.14 | 1.85 | 0.56 | 0.15 | 2.16 |
| Cold sores/oral herpes | No | 111 | 278 | 1.00 | | | 1.00 | | |
| | Yes | 7 | 17 | 0.98 | 0.38 | 2.50 | 0.70 | 0.25 | 1.99 |
| Any other infection[b] | No | 110 | 287 | 1.00 | | | 1.00 | | |
| | Yes | 9 | 15 | 1.45 | 0.59 | 3.57 | 1.45 | 0.55 | 3.82 |

[a]Several other specific infections were asked about. For each of them, fewer than three mothers in the whole sample said they had the infection (rubella, measles, chicken pox, shingles, mumps, glandular fever, pneumonia, hepatitis B, malaria, and leptospirosis). [b]'Other infections' are infections other than those in the Table or in (a) above. [c]The other variables in the multivariate models were: child's social class, household crowding, mother's educational level.

**Table 3** Odds ratios for childhood leukaemias in relation to certain infections of the child

| Infection[a] | Categories | No. of cases | No. of controls | Adjusted for age in years and sex only: Odds ratio | 95% CI Bounds: Lower | 95% CI Bounds: Upper | Adjusted for age, sex, and other variables[c]: Odds ratio | 95% CI Bounds: Lower | 95% CI Bounds: Upper |
|---|---|---|---|---|---|---|---|---|---|
| *Infections of the child at any time prior to 3 months before the reference date* | | | | | | | | | |
| Glandular fever | No | 119 | 302 | 1.00 | | | 1.00 | | |
| | Yes | 2 | 1 | 6.75 | 0.51 | 88.72 | 9.06 | 0.60 | 135.74 |
| Cold sores | No | 115 | 286 | 1.00 | | | 1.00 | | |
| | Yes | 5 | 15 | 0.86 | 0.29 | 2.51 | 1.10 | 0.34 | 3.58 |
| Giardiasis | No | 120 | 301 | 1.00 | | | 1.00 | | |
| | Yes | 1 | 2 | 1.05 | 0.09 | 12.53 | 0.46 | 0.03 | 6.37 |
| *Infections of the child in the first yr of life*[b] | | | | | | | | | |
| Whooping cough | No | 114 | 264 | 1.00 | | | 1.00 | | |
| | Yes | 2 | 3 | 1.48 | 0.23 | 9.45 | 3.90 | 0.43 | 35.10 |
| Measles | No | 111 | 256 | 1.00 | | | 1.00 | | |
| | Yes | 5 | 12 | 1.13 | 0.37 | 3.43 | 1.49 | 0.41 | 5.47 |
| Rubella | No | 115 | 264 | 1.00 | | | 1.00 | | |
| | Yes | 1 | 4 | 0.49 | 0.05 | 4.62 | 0.77 | 0.06 | 9.52 |
| Chicken pox | No | 109 | 256 | 1.00 | | | 1.00 | | |
| | Yes | 7 | 12 | 1.69 | 0.61 | 4.71 | 0.97 | 0.27 | 3.53 |
| Mouth infection | No | 104 | 246 | 1.00 | | | 1.00 | | |
| | Yes | 12 | 22 | 1.24 | 0.58 | 2.67 | 1.12 | 0.46 | 2.72 |
| Eye infection | No | 105 | 238 | 1.00 | | | 1.00 | | |
| | Yes | 11 | 30 | 0.75 | 0.35 | 1.58 | 0.56 | 0.23 | 1.39 |
| Ear infection | No | 80 | 192 | 1.00 | | | 1.00 | | |
| | Yes | 36 | 76 | 1.10 | 0.67 | 1.80 | 1.05 | 0.59 | 1.85 |
| Influenza | No | 107 | 263 | 1.00 | | | 1.00 | | |
| | Yes | 9 | 4 | 5.58 | 1.57 | 19.83 | 6.80 | 1.81 | 25.66 |
| Colds | No | 56 | 137 | 1.00 | | | 1.00 | | |
| | Yes | 60 | 131 | 1.05 | 0.66 | 1.65 | 1.10 | 0.64 | 1.89 |
| Persistent cough | No | 109 | 251 | 1.00 | | | 1.00 | | |
| | Yes | 6 | 17 | 0.69 | 0.26 | 1.84 | 0.80 | 0.26 | 2.45 |
| Diarrhoea and vomiting | No | 101 | 222 | 1.00 | | | 1.00 | | |
| | Yes | 15 | 46 | 0.62 | 0.33 | 1.19 | 0.53 | 0.25 | 1.12 |
| 'Other' infection | No | 99 | 243 | 1.00 | | | 1.00 | | |
| | Yes | 17 | 25 | 1.54 | 0.78 | 3.01 | 1.53 | 0.69 | 3.38 |
| Number of above types of infection in the first year of life | None | 22 | 70 | 1.00 | | | 1.00 | | |
| | One | 38 | 90 | 1.25 | 0.66 | 2.34 | 1.26 | 0.61 | 2.62 |
| | Two | 33 | 60 | 1.64 | 0.84 | 3.19 | 1.33 | 0.62 | 2.84 |
| | Three or more | 23 | 48 | 1.30 | 0.63 | 2.68 | 1.30 | 0.57 | 2.96 |
| Child had any infection in the first year of life | No | 22 | 70 | 1.00 | | | 1.00 | | |
| | Yes | 94 | 198 | 1.37 | 0.78 | 2.41 | 1.29 | 0.68 | 2.46 |

[a]Several other infections were asked about, but fewer than two mothers in the whole sample said the child had the infection (hepatitis B, poliomyelitis, and cytomegalovirus infection prior to 3 months before the reference date; and mumps in the first year of life). [b]For analyses of infections in the first year of life, cases and controls aged under 15 months on their reference dates were excluded. [c]The other variables in the multivariate models were: child's social class, household crowding, residential mobility, interview year and delay from reference date to interview.

*© 1999 Cancer Research Campaign*

**Table 4** Odds ratios for childhood leukaemias in relation to the child's vaccinations

| Vaccination | Categories | No. of cases[a] | No. of controls[a] | Adjusted for age in years and sex only: Odds ratio | 95% CI Bounds: Lower | 95% CI Bounds: Upper | Adjusted for age, sex, and other variables[b]: Odds ratio | 95% CI Bounds: Lower | 95% CI Bounds: Upper |
|---|---|---|---|---|---|---|---|---|---|
| Had all usual immunizations? | No | 5 | 25 | 1.00 | | | 1.00 | | |
| (if 'no' & due to cancer, excluded) | Yes | 94 | 268 | 2.10 | 0.75 | 5.83 | 2.38 | 0.73 | 7.74 |
| Had any 'other' vaccinations before | No | 117 | 291 | 1.00 | | | 1.00 | | |
| reference date? | Yes | 2 | 6 | 0.93 | 0.18 | 4.91 | 0.63 | 0.06 | 6.26 |
| Triple vaccine[c] | No | 38 | 88 | 1.00 | | | 1.00 | | |
| | Yes | 80 | 197 | 0.73 | 0.44 | 1.20 | 1.01 | 0.52 | 1.95 |
| Double vaccine | No | 57 | 151 | 1.00 | | | 1.00 | | |
| | Yes | 57 | 132 | 0.84 | 0.52 | 1.37 | 0.82 | 0.44 | 1.54 |
| Polio sip | No | 37 | 87 | 1.00 | | | 1.00 | | |
| | Yes | 80 | 197 | 0.74 | 0.45 | 1.23 | 0.90 | 0.47 | 1.74 |
| Hepatitis B vaccine | No | 52 | 131 | 1.00 | | | 1.00 | | |
| | Yes | 62 | 151 | 0.69 | 0.41 | 1.17 | 0.93 | 0.49 | 1.76 |
| Measles vaccine | No | 55 | 166 | 1.00 | | | 1.00 | | |
| | Yes | 58 | 106 | 1.52 | 0.95 | 2.45 | 1.87 | 1.00 | 3.48 |
| MMR vaccine | No | 112 | 270 | 1.00 | | | 1.00 | | |
| | Yes | 6 | 15 | 0.73 | 0.27 | 2.00 | 0.80 | 0.26 | 2.42 |
| Rubella (alone) vaccine | No | 114 | 257 | 1.00 | | | 1.00 | | |
| | Yes | 1 | 5 | 0.78 | 0.08 | 7.71 | 0.50 | 0.03 | 8.10 |
| BCG Mantoux testing | No | 118 | 295 | 1.00 | | | 1.00 | | |
| | Yes | 2 | 2 | 2.08 | 0.27 | 15.95 | 1.74 | 0.19 | 15.86 |
| Polio booster | No | 112 | 279 | 1.00 | | | 1.00 | | |
| | Yes | 5 | 12 | 0.99 | 0.32 | 3.04 | 0.97 | 0.26 | 3.59 |
| BCG vaccine | No | 112 | 284 | 1.00 | | | 1.00 | | |
| | Yes | 8 | 11 | 1.55 | 0.60 | 4.05 | 1.05 | 0.33 | 3.32 |
| Other vaccines | No | 117 | 280 | 1.00 | | | 1.00 | | |
| | Yes | 2 | 17 | 0.20 | 0.05 | 0.92 | 0.21 | 0.04 | 1.12 |
| No. of different vaccinations[c] | None | 37 | 78 | 1.00 | | | 1.00 | | |
| | One or two | 3 | 19 | 0.26 | 0.07 | 0.95 | 0.52 | 0.12 | 2.23 |
| | Three or four | 34 | 111 | 0.55 | 0.30 | 0.98 | 0.57 | 0.27 | 1.21 |
| | Five or more | 44 | 79 | 0.80 | 0.44 | 1.46 | 0.99 | 0.45 | 2.18 |
| Any vaccinations? | No | 37 | 78 | 1.00 | | | 1.00 | | |
| | Yes | 81 | 209 | 0.62 | 0.37 | 1.03 | 0.71 | 0.36 | 1.38 |

[a]Children aged under 3 months on the reference date were excluded from the analyses for: 'all usual immunizations', 'any other vaccinations', triple, double, hepatitis B, BCG and 'other' vaccines; and BCG Mantoux testing. Children aged under 6 months on the reference date were excluded from the analyses for polio sip and polio booster. Children under 9 months on the reference date were excluded from the analyses for measles and MMR vaccines, no. of vaccines, and 'any vaccinations'. Children aged under 15 months on the reference date were excluded from the analyses for rubella (alone) vaccine. [b]The other variables in the multivariate models were: child's social class, child's ethnic group, mother's marital status, mother's education, mother's home ownership, household crowding, delay from reference date to interview, interview year. [c]Specific vaccinations (triple, double, polio etc), including those contributing to the last two variables in the table, were only counted if the year in which they were given was known and was before the reference year.

In the unmatched analyses, age (in single years) and sex were always adjusted for. Other potential confounders were listed for each exposure or group of exposures. They were then tested to see whether they confounded the estimates for key variables chosen to represent each group of exposures. A potential confounder was adjusted for if adding it to the model (for any key variable) changed the age- and sex-adjusted odds ratio for combined leukaemias by more than 10% (Maldonado and Greenland, 1993).

### Inter-method reliability

The parents of 113 of the 121 cases (93%) and 111 of the 121 matched comparison children (92%) gave us permission to contact their general practitioners (GPs), who were sent brief standardized questionnaires about selected exposures. These were completed by GPs of 93 cases (77%) and 87 matched controls (72%). The comparison was restricted to the 124 (70 cases, 54 controls) whose GPs' records began before the children were aged 18 months.

### Serology

If consent was given, hospital workers took venipuncture or finger-prick samples from cases (if possible before treatment), and interviewers took finger-prick samples from controls. Sera were sent to the NZ Communicable Disease Centre (NZCDC) for the following assays: measles immunoglobulin G (IgG), cytomegalovirus IgG, Epstein–Barr virus capsid antigen (VCA) IgG, and poliomyelitis virus (types 1–3) antibody titres. Analyses were based on pretreatment results. Serological testing was conducted for 116 (96%) of the 121 cases (but only 41 (34%) had pretreatment samples) and for 127 (42%) of the 303 controls (interviewers had difficulties obtaining enough blood from controls). Antibody levels were categorized as suggested a priori by the NZCDC. Measles, Epstein–Barr virus and cytomegalovirus antibody results were reported as an index and categorized as: > 1.1 (positive), < 0.9 (negative) and 0.9–1.1 (equivocal). Poliomyelitis antibody titres were categorized as: < 8 (no detectable antibodies), 8–32 (low antibody response), 64–256 (moderate) and 512 to > 1024 (high). Because there were

 *© 1999 Cancer Research Campaign*

**Table 5** Odds ratios for acute lymphoblastic leukaemia in relation to various factors indirectly related to exposure to infections

| | | | | Adjusted for age in years and sex only | | | Adjusted for age, sex, and other variables[a] | | |
|---|---|---|---|---|---|---|---|---|---|
| | | | | | 95% CI Bounds | | | 95% CI Bounds | |
| Factor/exposure | Categories | No. of cases | No. of controls | Odds ratio | Lower | Upper | Odds ratio | Lower | Upper |
| How many houses did child live in before reference date? | None or One | 32 | 137 | 1.00 | | | 1.00 | | |
| | Two | 32 | 92 | 1.47 | 0.82 | 2.66 | 1.48 | 0.81 | 2.72 |
| | Three or more | 33 | 74 | 2.52 | 1.31 | 4.85 | 2.36 | 1.19 | 4.67 |
| Household crowding: average no. of people per room in house child lived in at birth | ≤0.50 | 18 | 35 | 1.00 | | | 1.00 | | |
| | >0.50 to 1.00 | 60 | 224 | 0.49 | 0.25 | 0.97 | 0.44 | 0.21 | 0.92 |
| | >1.00 | 16 | 40 | 0.87 | 0.36 | 2.06 | 0.61 | 0.22 | 1.64 |
| Did child share a bedroom in house lived in at birth? | No | 24 | 105 | 1.00 | | | 1.00 | | |
| | Yes | 70 | 194 | 1.36 | 0.79 | 2.35 | 1.15 | 0.65 | 2.03 |
| Was index child the mother's firstborn? | No | 60 | 197 | 1.00 | | | 1.00 | | |
| | Yes | 37 | 106 | 1.09 | 0.66 | 1.80 | 1.09 | 0.66 | 1.80 |
| Did mother live on farm during the pregnancy or 3 months before? | No | 84 | 245 | 1.00 | | | 1.00 | | |
| | Yes | 13 | 58 | 0.64 | 0.32 | 1.24 | 0.77 | 0.38 | 1.55 |
| Did child ever live on a farm for more than 1 month before reference date? | No | 83 | 231 | 1.00 | | | 1.00 | | |
| | Yes | 14 | 68 | 0.62 | 0.32 | 1.20 | 0.75 | 0.38 | 1.48 |
| Did child have regular contact with other children from outside home at <12 months[b] | No | 39 | 94 | 1.00 | | | 1.00 | | |
| | Yes | 51 | 172 | 0.67 | 0.40 | 1.12 | 0.65 | 0.36 | 1.17 |
| Did mother breastfeed the child? | No | 8 | 34 | 1.00 | | | 1.00 | | |
| | Yes | 89 | 269 | 1.10 | 0.47 | 2.56 | 0.98 | 0.39 | 2.47 |
| Age child was when mother gave last breastfeed | Child not breastfed | 8 | 34 | 1.00 | | | 1.00 | | |
| | 2 days to 6 months | 47 | 123 | 1.35 | 0.56 | 3.27 | 1.24 | 0.47 | 3.23 |
| | >6 months to 1 year | 25 | 84 | 0.88 | 0.35 | 2.25 | 0.82 | 0.29 | 2.27 |
| | over 1 year | 15 | 62 | 0.73 | 0.27 | 1.98 | 0.47 | 0.15 | 1.43 |
| Age child was when solid food was first introduced | 4 months or younger | 44 | 180 | 1.00 | | | 1.00 | | |
| | older than 4 months | 48 | 122 | 1.60 | 0.98 | 2.61 | 1.25 | 0.73 | 2.12 |
| How did mother sterilize child's bottles/utensils? | Didn't sterilize them | 24 | 82 | 1.00 | | | 1.00 | | |
| | Boiling | 38 | 107 | 1.39 | 0.75 | 2.57 | 1.39 | 0.75 | 2.57 |
| | Solution or tablets | 35 | 108 | 1.28 | 0.69 | 2.37 | 1.28 | 0.69 | 2.37 |
| Did child ever have a blood transfusion before reference date?[c] | No | 94 | 299 | 1.00 | | | 1.00 | | |
| | Yes | 3 | 3 | 2.30 | 0.42 | 12.64 | 2.54 | 0.35 | 18.30 |

[a]The other variables in the multivariate models were as follows: 'residential mobility': mother's marital status, child's infection with influenza in the first year of life; 'household crowding': child's social class, mother's marital status, mother's education, child's infection with influenza in the first year of life, age child was when solid food was first introduced; 'bedroom sharing': child's social class, mother's education; 'firstborn': no other variables; 'mother living on a farm in pregnancy': child's social class, mother's marital status; for 'child living on a farm': child's social class; 'regular contact with other children': child's social class, mother's marital status, mother's educational level, mother's smoking during pregnancy, household crowding, residential mobility, and delay from reference date to interview; 'breastfeeding' and 'age stopped breastfeeding': child's social class, mother's education, household crowding, delay from reference date to interview; 'age when solid food introduced': household crowding, delay from reference date to interview; 'method of sterilization of bottles/utensils': no other factors; 'blood transfusion': child's social class, mother's home ownership, mother's education, delay from reference date to interview. [b]In the analyses for 'regular contact with other children from outside the home at under 12 months of age', cases and controls who were aged less than 15 months on their reference dates were excluded. [c]Transfusions within 3 months of the reference date are excluded.

fewer children in the serological analyses, a 20% cut-off was used to select confounders.

### RESULTS

Risks of both combined leukaemias and ALL were significantly increased among children from the 'no paid jobs–not classifiable' social class group, and significantly decreased among those whose mothers were married (Table 1). There were differences related to the mother's education ($P = 0.001$); those whose mothers had no qualifications had the highest risks. For ALL, there was a significant decrease in risk associated with high gestational age, and a significant increase with maternal smoking in pregnancy.

We found no statistically significant associations of childhood leukaemia with maternal infections during the pregnancy or 3 months before (Table 2), or with certain infections of the child at any time prior to 3 months before the reference date (Table 3). Risk was significantly increased for reported influenza infection in the first year of life (adjusted odds ratio (OR) 6.8 (95% confidence interval (CI) 1.8–25.7)). No associations were found with other infections in the first year of life (Table 3), or with the number of different types of infection. The findings for ALL (not shown) were similar to those for combined leukaemias; for reported influenza the adjusted OR was 6.0 (1.4–26.2).

Measles vaccine (given alone) showed a raised leukaemia OR of borderline significance (1.9, 95% CI 1.0–3.5; Table 4). For

© 1999 Cancer Research Campaign 

Measles/Mumps/Rubella (MMR), the OR was 0.8. Although there were some significant findings for intermediate categories of the number of different vaccinations of the child, the ORs moved towards 1.0 and lost significance with adjustment for other confounders. There was no significant trend with the number of different vaccinations ($P = 0.84$). The ORs for ALL (not shown) were similar.

For ALL there was a positive trend ($P = 0.01$) in relation to residential mobility (Table 5). The OR for household crowding was significantly reduced for the middle category, but there was no trend ($P = 0.28$). There was a trend in ALL risk with increasing duration of breastfeeding ($P = 0.04$); those breastfed for more than a year had the lowest risk (OR 0.5). No significant associations were found with attendance at particular types of preschool groups in the first year of life (results not shown). There were no significant associations between leukaemia and living with pets (dogs, cats, other furry animals or birds) during the pregnancy or the 3 months before (mother), or before the reference date (child)-adjusted results, not shown.

### Adjusted ALL results from unconditional versus conditional logistic regression

The unmatched and matched analyses gave similar results for ALL (within the bounds of statistical variation). The confidence intervals from the matched were usually wider. Influenza in the first year was associated with a non-significant increase in ALL risk in the conditional logistic regression (OR 9.1, 95% CI 0.9–88.7). The variables showing significant or borderline associations in the conditional logistic regression were eye infections in the first year of life (OR 0.2, 95% CI 0.1–0.7), residential mobility (highest category OR 2.3, 95% CI 1.0–4.9), household crowding (middle category OR 0.3, 95% CI 0.1–1.0; highest category OR 0.4, 95% CI 0.1–1.8), and age at which solids were introduced (> 4 vs ≤ 4 months OR 1.9, 95% CI 1.0–3.5).

### Comparison of information from mothers and GPs

For birth weight, MMR vaccination, measles infection and mouth infection, the percentage agreement was 80% or more for both cases and controls. For measles vaccination, polio vaccination, ear infection, upper respiratory tract infection and diarrhoea and vomiting, the agreement was poor (below 80%) for controls and/or cases. For each variable, the percentage agreement did not differ significantly between cases and controls ($\chi^2$ with Yates' correction).

### Serology

The adjusted childhood leukaemia OR for poliovirus type 1 serology ('high' vs 'no or low' titres) was 14.6 (95% CI 1.6–136.1). The results for measles, Epstein–Barr virus, cytomegalovirus and polioviruses types 2 and 3 showed no significant associations. Further details are available elsewhere (Dockerty, 1997).

### DISCUSSION

There was little evidence of associations between childhood leukaemia and infections; the exception being a statistically significant increase in risk with reported influenza in the first year of life (OR 6.8, 95% CI 1.8–25.7). This may be a chance association due to multiple comparisons. 'Influenza' was specified on prompt cards used by the interviewers. It could be confused with other viruses or be asymptomatic, but such misclassification should be non-differential, moving the OR towards 1.0. For seven of the nine exposed cases and three of the four exposed controls, the mothers reported that the GP was consulted. This high consultation rate is consistent with the diagnosis. But even for GPs, diagnosis can be difficult.

We checked for significant bias due to non-participation or recall using two variables from the birth registrations: the parents' marital status (recording of date of marriage) and the paternal occupation (whether or not it was given; unemployed and retired were counted as no occupation). Relevant information was available for 129 of 131 eligible cases. There was no difference in the percentage married (68% of interviewed and 67% of eligible) or in the percentage giving no paternal occupation (13% interviewed and 14% eligible). For the controls, the 94 non-interviewed first-choice controls were compared with the 94 replacements (whose parents were interviewed). Significant differences were found. The percentages married were 65% (non-interviewed first-choice), and 94% (interviewed replacement controls). The percentages with no listed paternal occupation were 13% (non-interviewed first-choice) and 3% (interviewed replacements). Logistic regressions were conducted using the birth information from the eligible cases (129 with information) and the 303 eligible first-choice controls. In the eligible population, the risk of leukaemia was decreased for children whose mothers were married (age- and sex-adjusted OR 0.5, 95% CI 0.3–0.9); and increased for children whose birth records gave no paternal occupation (age- and sex-adjusted OR 2.0, 95% CI 1.1–4.0). Each association was in the same direction as the associations found among the participants.

Thus, even when the bias due to non-participation was eliminated for these variables (and recall bias could not have occurred), the same kinds of associations were found. This increases confidence in the main results for childhood leukaemia related to marital status and social class. The differences between the eligible population and the participants are unlikely to be important enough to account for the observed strong association with influenza, which persisted after controlling for confounders such as residential mobility and social class. However, confounding by an unknown strong risk factor cannot be ruled out. The association with reported influenza was based on small numbers of exposed children and there were multiple comparisons, so it may be due to chance. It was not specific to leukaemias, because analyses comparing children with combined solid cancers and the controls also showed an association with reported influenza in the first year of life (age- and sex-adjusted OR 4.0 (1.2–14.0); multiple variable-adjusted OR 4.1 (1.1–15.2)). This could indicate a low estimate of the exposure prevalence in the control group, due to chance variation or bias. A causal interpretation may be less likely, but cannot be ruled out.

No previous case-control or cohort studies have reported on influenza infections of the child. In a cohort study, Fedrick and Alberman (1972) found a strong positive association between influenza of the mother during the pregnancy and leukaemia or lymphoma of the child up to age 11 years (relative risk 9, $P < 0.001$). In the Oxford Survey of Childhood Cancers, a case-control study, there was an increased risk of combined childhood cancers in relation to influenza in pregnancy (OR 1.5, 95% CI 1.1–2.1), but no association for leukaemias separately (Bithell et al, 1973). The comparability of these with the current study depends on whether

*© 1999 Cancer Research Campaign*

influenza infections in-utero and in early childhood could have similar effects. There are several mechanisms by which particular viruses can cause cancer (Dimmock and Primrose, 1994), but there is no more direct evidence of a carcinogenic potential of influenza viruses. The current finding for reported influenza needs further testing in analytical studies elsewhere.

The small increase in leukaemia risk with measles vaccination is of borderline statistical significance (OR 1.9, 95% CI 1.0–3.5). This is likely to be a chance finding because the odds ratios for MMR vaccination and measles serology were below 1.0.

### Greaves' hypothesis

Greaves' (1988) hypothesis predicted an increased risk of B-cell precursor ALL for those of higher socioeconomic level, firstborn children, and those with delayed or diminished exposure to common infections in infancy (Greaves, 1988; Neglia et al, 1990). Prolonged breastfeeding, day-care attendance, crowding in houses and timely completion of early childhood immunizations were predicted to decrease the risk of B-precursor ALL (Greaves, 1988; Neglia et al, 1990; Greaves and Alexander, 1993). Greaves' (1988) hypothesis is not consistently supported by our results. In particular, the socioeconomic findings for ALL were in the opposite direction to that predicted, even in the eligible population. There was no association between ALL and firstborn status (OR 1.1), number of different infections in the first year of life (≥ 3 infections, OR 1.3) or attendance at preschool groups. The OR for 'any regular contact with children from outside the home' (0.7) was in the direction predicted, but not statistically significant. The findings for the highest categories of household crowding and duration of breastfeeding were in the direction predicted. There was no association with the number of vaccinations (≥ 5 vaccinations, ALL OR 1.1). Analyses restricted to the 87 cases with B-cell precursor ALL showed very similar results (not shown). However, a larger study would be needed to find a small effect or an effect limited to certain subgroups.

### Residential mobility

There was a significant positive association with residential mobility. Of the 303 eligible first-choice controls, 46 (15%) did not participate because they could not be traced. They almost certainly moved houses, and residential mobility among the controls would be underestimated. Therefore, this association is probably spurious, and should not be regarded as evidence supporting Kinlen's hypothesis.

### Poliovirus serology

Although there was an increased risk of leukaemia in relation to poliovirus type 1 serology, there was no clear dose–response and no association with oral poliomyelitis vaccine (questionnaire). Low percentages of cases and controls contributed serological information, and the smaller numbers meant that confounder control was limited. The increased risk for poliovirus type 1 may be due to residual confounding or chance variation.

### CONCLUSION

This study gives little support to hypotheses of an infective aetiology for childhood leukaemia. The raised odds ratio for reported influenza infection in the first year of life could be real, or due to chance variation. The questions about infectious exposures were based on those of the large UK childhood cancer study, which should soon produce results.

### ACKNOWLEDGEMENTS

We are grateful to all the families who took part, and to: *Interviewers*: J Adams-Schneider, P Babe, C Brown, P Carr, J Cottrell, J Egan, V Leigh, J Ross, Jill Sye and Joan Sye; *Secretaries and Research Assistants:* E Andrews, S McCord, S Paulin, M Stevens and J Ward; *NZCDC:* J Miller and P Raynel; *NZ Cancer Registry*: J Fraser, L Mooney and B Wordsworth; *Pathologists*: B Berkeley, Y Chan, B Synek and L Teague; *Clinicians*: G Abbott, D Holdaway, S Macfarlane, D Mauger, B Robinson, J Skeen and teams; *Others* (for contributions or advice): JL Dockerty, Sir R Doll, P McKinney, C Manning, E Roman, K Sharples, J Smeijers and G Spears. This study was funded by Health Research Council of NZ, NZ Lottery Grants Board, A.B. de Lautour Charitable Trust, Otago Medical School, Transpower NZ, Cancer Society of NZ, Otago Medical Research Foundation and Variety Handcrafts.

#### REFERENCES

Bithell JF, Draper GJ and Gorbach PD (1973) Association between malignant disease in children and maternal virus infections. *Br Med J* **1**: 706–708

Breslow NE and Day NE (1980) *Statistical Methods in Cancer Research, Vol 1 – The Analysis of Case-Control Studies*. International Agency for Research on Cancer: Lyon

Dimmock NJ and Primrose SB (1994) *Introduction to Modern Virology*, 4th edn. Blackwell Science: Oxford

Dockerty JD (1997) The epidemiology of childhood leukaemia in New Zealand, with particular reference to hypotheses related to an infective cause. University of Otago, Dunedin, New Zealand. pp. 1–535. Thesis for the degree of Doctor of Philosophy.

Dockerty JD, Elwood JM, Skegg DCG and Herbison GP (1998) Electromagnetic field exposures and childhood cancers in New Zealand. *Cancer Causes Control* **9**: 299–309

Fedrick J and Alberman ED (1972) Reported influenza in pregnancy and subsequent cancer in the child. *Br Med J* **2**: 485–488

Greaves MF (1988) Speculations on the cause of childhood acute lymphoblastic leukemia. *Leukemia* **2**: 120–125

Greaves MF and Alexander FE (1993) An infectious etiology for common acute lymphoblastic leukaemia in childhood? *Leukemia* **7**: 349–360

Hamajima N, Hirose K, Inoue M, Takezaki T, Kuroishi T and Tajima K. (1994) Case-control studies: matched controls or all available controls? *J. Clin. Epidemiol.* **47**: 971–975.

Kinlen L (1988) Evidence for an infective cause of childhood leukaemia: comparison of a Scottish new town with nuclear reprocessing sites in Britain. *Lancet* **ii**: 1323–1327

Kinlen LJ (1996) Epidemiological evidence for an infective basis in childhood leukaemia. *J. Roy. Soc. Health* **116**: 393–399

Kinlen LJ (1997) High-contact paternal occupations, infection and childhood leukaemia: five studies of unusual population-mixing of adults. *Br. J. Cancer* **76**: 1539–1545

Maldonado G and Greenland S. (1993) Simulation study of confounder-selection strategies. *Am. J. Epidemiol.* **138**: 923–936.

Neglia JP, Severson RK and Linet MS (1990) A reply to MF Greaves: toward a testable etiology of childhood acute lymphoblastic leukemia. *Leukemia* **4**: 517–521.

Office of Population Censuses and Surveys (1991) *Standard Occupational Classification, Vol 3 – Social Classifications and Coding Methodology*. HMSO: London

Rothman KJ (1986) *Modern Epidemiology*. Little, Brown: Boston

*© 1999 Cancer Research Campaign*
